# Mechanisms of Changma Xifeng tablet in alleviating Tourette syndrome via modulation of neurotransmitters, inflammatory responses, and metabolic pathways

**DOI:** 10.3389/fpsyt.2026.1773753

**Published:** 2026-04-10

**Authors:** Yuanyang Shao, Chang-E Guo, Kun Gao, Meng Bian, Lili Li, Lingyan Zhang, Mengjuan Wu, Juan Wang, Chunsheng Zhu

**Affiliations:** 1 Department of Chinese Medicine, The First Affiliated Hospital of Zhengzhou University, Zhengzhou, China; 2 Pharmacy Department, Beijing Fengtai Hospital of Chinese Medicine (Beijing Fengtais Hospital of Nanyuan District), Beijing, China

**Keywords:** Changma xifeng tablet, lipid metabolism, neurotransmitters, serum metabolomic, Tourette syndrome

## Abstract

**Introduction:**

Tourette syndrome (TS) is a common pediatric neurodevelopmental disorder. Changma Xifeng tablet (CMXF), a traditional Chinese medicinal formulation, is widely used clinically for TS but its mechanisms remain unclear. This study aimed to evaluate CMXF’s efficacy and explore its action mechanisms via a TS murine model.

**Methods:**

The chemical constituents of CMXF were analyzed and identified by UPLC-Q-TOF-MS/MS. Sixty male BALB/c mice were divided into 6 groups (CON, MOD, HAL, CMXF-L/M/H). TS models were induced by IDPN injection. Behavioral assessments, ELISA (neurotransmitters/inflammatory cytokines), HE staining (striatal pathology), RT-qPCR/WB (DRD1/DRD2/COMT/MAO-B), and UHPLC-Q-TOF LC-MS (serum metabolomics) were performed.

**Results:**

A total of twenty-one components, including gallic acid, gastrodin, catechin, sibiricose A3, paeoniflorin, parishin B, and tenuifoliside B, were identified in CMXF. From week 4 to week 8, the HAL group and CMXF-H group showed significantly improved behavioral performance compared with the MOD group (*F* > 1, *P* < 0.001), presenting a dose-dependent trend. ELISA results revealed that CMXF-H significantly increased the levels of 5-HT in serum and striatum (*F* > 1, *P* < 0.001), while decreasing the levels of DA, HVA, NE, IL-1β, and IL-6 in serum (*F* > 1, *P* < 0.001). RT-qPCR and WB results indicated that CMXF regulated the mRNA and protein, inhibiting DRD1/DRD2 expressions and inducing COMT/MAO-B expressions. Serum metabolomics analysis identified 398 differentially expressed metabolites, which were mainly involved in lipid metabolism and organic acid metabolism pathways.

**Discussion:**

CMXF exerts anti-TS effects by regulating neurotransmitters, inflammatory cytokines, DA signaling, and serum metabolites, providing novel insights into its mechanisms and potential targeted therapy for TS.

## 1 Introduction

Tourette syndrome (TS) (1) is a complex neuropsychiatric disorder with onset typically in childhood, characterized by involuntary, sudden, and repetitive motor tics that can affect multiple body regions (2). Common manifestations include motor tics (e.g., facial tics: blinking, frowning, lip puckering; head tics: nodding, shaking; limb tics: shrugging, arm flinging, kicking) and, in a subset of patients, vocal tics (e.g., throat clearing, coughing, grunting, or rarely coprolalia) (3). Epidemiological studies estimate the global prevalence of TS at approximately 1%.

Current therapeutic approaches for TS include pharmacotherapy, psychotherapy, and—for refractory cases—surgical interventions, though the latter is rarely employed due to its invasiveness (4, 5). Pharmacological management often involves agents such as methylphenidate, atomoxetine, and amphetamines (6). These drugs primarily enhance central dopamine (DA) and norepinephrine (NE) signaling to modulate executive function and attention, domains frequently impaired in TS (7). Specifically, amphetamines act by inhibiting the dopamine transporter (DAT) and norepinephrine transporter (NET), suppressing vesicular monoamine transporter 2 (VMAT-2), and reducing monoamine oxidase activity; methylphenidate primarily inhibits DAT and NET, while also modulating serotonin 1A receptor activity and redistributing VMAT-2 (8). Despite their clinical efficacy in alleviating tics and associated symptoms, these medications are associated with adverse effects (e.g., insomnia, anxiety) and carry abuse potential, limiting their long-term utility in some pediatric populations (9).

In contrast, herbal medicines and traditional Chinese medicine (TCM) formulations have shown therapeutic potential for various central nervous system disorders, with advantages including lower addiction risk, milder side effects, and gradual onset of action—factors that may reduce adverse reaction rates and improve treatment adherence (10). In TCM theory, TS is classified under “liver wind” and “chronic convulsive wind” disorders. Therapeutic principles emphasize calming the liver to suppress wind, resolving phlegm, and restoring visceral balance (11). Changma Xifeng tablet (CMXF), a widely used TCM formulation for TS, aligns with these principles. It comprises five herbal components: *Paeoniae Radix Alba* (monarch herb), *Acori Tatarinowii Rhizoma*, *Gastrodiae Rhizoma* (adjuvant herbs), *Margaritifera Concha*, and *Polygalae Radix* (assistant herbs) (11, 12). As the core “monarch herb”, *Paeoniae Radix Alba* is traditionally valued for nourishing blood, astringing yin to arrest sweating, soothing the liver to alleviate pain, and calming liver yang; modern studies have further confirmed its anti-inflammatory, analgesic, and hepatoprotective activities (13). The adjuvant and assistant herbs synergistically enhance effects of calming the liver, suppressing wind, tranquilizing the mind, and resolving phlegm in TCM framework (14).

While previous studies on CMXF for TS have largely emphasized its clinical efficacy, mechanistic insights into its anti-TS properties remain limited (15). This gap constrains a deeper understanding of its pharmacological foundation and rational clinical use. To bridge this knowledge deficit, we employed a murine TS model and an integrated experimental strategy to systematically evaluate the therapeutic potential of CMXF. Our approach combined longitudinal behavioral analyses—assessing stereotyped movements, spontaneous activity, spatial restriction behaviors, and head–body twitch frequency over 8 weeks—with histopathological examination of striatal tissues via HE staining, quantification of neurotransmitters (5-HT, DA, HVA, NE) and inflammatory cytokines (interleukin-1β (IL-1β), interleukin-6 (IL-6) in serum and striatum using ELISA, serum metabolomics profiling by UHPLC-Q-exactive LC-MS, and RT-qPCR and Western Blot (WB) analyses of key molecular targets DA receptors 1 and 2(DRD1 and DRD2), catechol-O-methyltransferase (COMT), and monoamine oxidase B (MAO-B). Through this multi-level investigation, we aimed to clarify the potential mechanisms by which CMXF ameliorates TS, thereby contributing a more solid scientific basis for its clinical application.

## 2 Materials and methods

### 2.1 Materials

CMXF tablet was purchased from JiRen Pharmaceutical (National medical product approval number Z20140013, China). Haloperidol was purchased from Ningbo Dahongying Pharmaceutical (National medical product approval number H33020585, China). 3,3’-iminodipropionitrile (IDPN) solution (cat. no. 317306) was purchased from Sigma-Aldrich (St. Louis, MO, USA). ELISA kits for detecting serotonin/5-hydroxytryptamine (5-HT, cat. no. E-EL-0033), dopamine (DA, cat. no. E-EL-0046), and NE (cat. no. E-EL-0047) were obtained from Elabscience Biotechnology Co., Ltd. (Wuhan, China). The mouse homovanillic acid (HVA) ELISA kit (cat. no. FT-P9S735X) was purchased from Shanghai Fantai Biological Technology Co., Ltd. (Shanghai, China), while mouse interleukin-1*β* (IL-1*β*, cat. no. H-ELM0604b) and interleukin-6 (IL-6, cat. no. H-ELM0609b) ELISA kits were supplied by Henan Dinghan Biological Technology Co., Ltd. (Zhengzhou, China). All reagents were stored and used in strict accordance with the manufacturers’ instructions.

### 2.2 The identification of the compounds in CMXF by UPLC-Q-TOF-MS/MS

CMXF was crushed and weighed, with 0.1 g of powder added into 8 mL of 50% methanol. After shaking, the mixture was ultrasonicated for 30 min, and then subsequently centrifuged at 4°C and 14,000 rpm for 20 min. The supernatant was placed in the injection vial for analysis. The supernatant was analyzed on a Waters ACQUITY HSS T3 (2.1 mm×100 mm, 1.7 μm) column. The column temperature was maintained at 40°C, with a flow rate of 0.3 mL/min and an injection volume of 2.0 μL. The mobile phase A consisted of a 0.2% formic acid aqueous solution, while mobile phase B was acetonitrile. The gradient elution condition was detailed in supplementary **Supplementary Table 1** and **S2**. Data were acquired using an Agilent 1290 UPLC system coupled with a Q-TOF mass spectrometer equipped with an electrospray ionization (ESI) source in both positive and negative ion modes.

### 2.3 Animal experiments

Sixty specific pathogen-free (SPF)-grade male BALB/c mice (8–12 weeks old, 20 ± 2 g body weight) were purchased from SPF (Beijing) Biotechnology Co., Ltd. (Beijing, China; license No. SCXK (Jing) 2024-0001). Mice were housed in a controlled environment (temperature: 22 ± 2 °C, relative humidity: 50 ± 5%, 12 h light/dark cycle) with free access to standard rodent chow and sterile water, and acclimatized for 1 week prior to experimentation to minimize stress.

Mice were randomly divided into 6 groups (n=10 per group) using a random number generator: Except for the CON group, the remaining 50 mice were used to establish the TS model via intraperitoneal injection of IDPN solution at a dose of 350 mg/kg once a day for 7 days (16). Mice were fasted for 12 h before each injection, with free access to water maintained. Successful model establishment was confirmed by a significant increase in stereotyped behavior scores (assessed using a validated scoring system; **Supplementary Table 3**) compared to the CON group. After model validation, mice in each treatment group received daily gavage administration for 8 consecutive weeks. Control group (CON): No modeling, given 0.9% normal saline via gavage; TS model group (MOD): TS model established, given 0.9% normal saline via gavage; Positive control group (HAL): TS model established, given haloperidol (a clinical anti-TS drug, 0.8 mg/kg) via gavage; CMXF low/medium/high-dose group (CMXF-L/M/H): TS model established, given low (360 mg/kg)/medium (720 mg/kg)/high (1440 mg/kg) -dose CMXF via gavage. The administered dosages of HAL and CMXF was calculated according to the routine dosage of children in clinical practice.

This study was approved by the Life Science Ethics Committee of the First Affiliated Hospital of Zhengzhou University (approval no. 2023-KY-1436-001) and strictly adhered to the National Institutes of Health Guide for the Care and Use of Laboratory Animals.

### 2.4 Behavioral assessments

Longitudinal behavioral assessments were performed at 0, 2-, 4-, 6-, and 8-weeks post-administration to evaluate TS-like symptoms (17). For each assessment (18) (1): Mice were individually placed in a transparent observation cage (30×20×25 cm) and allowed a 5-minute adaptation period to acclimate to the novel environment (2); Two independent observers, unaware of group assignments (double-blind design), scored stereotyped movements (e.g., repetitive grooming, head shaking), spontaneous activity (total distance traveled in the cage), and spatial restriction behaviors (time spent in the central vs. peripheral area of the cage) using a validated 4-point scale (**Supplementary Table 3**) (3); The frequency of head-body twitches (a core TS-like phenotype) was manually counted within a 1-minute window. Inter-observer reliability was verified (intraclass correlation coefficient >0.90) to ensure consistency of scoring.

### 2.5 Sample collection

After 8 weeks of treatment, mice were euthanized for sample collection. Mice were weighed using an electronic balance (accuracy: 0.01 g) before euthanasia. Mice were anesthetized via intraperitoneal injection of 2% sodium pentobarbital (40 mg/kg body weight) to ensure deep anesthesia (confirmed by loss of pedal reflex). Blood was collected from the abdominal aorta using a heparinized syringe. Serum was separated by centrifugation at 3000×g for 10 min at 4 °C, aliquoted, and stored at -80 °C for subsequent ELISA and metabolomic analyses. The chest area of anesthetized mice was shaved and disinfected with 75% ethanol. The thorax was opened to expose the heart, and a 24-gauge perfusion needle was inserted into the left ventricle (advanced to the aortic root). The right atrium was incised to allow outflow, and 37 °C sterile physiological saline was perfused at a rate of 5 mL/min until a clear, colorless liquid flowed from the right atrium (to remove blood contaminants). This was followed by perfusion with 4% paraformaldehyde (PFA) at the same rate for 15 min to fix brain tissue. Mice were then decapitated, and the whole brain was removed. The striatum was dissected on ice using a brain matrix (coronal sections: -0.5 to -1.5 mm relative to bregma, based on the mouse brain atlas) and divided into two portions: one was stored at -80 °C for ELISA, RNA, and protein extraction; the other was fixed in 4% PFA at room temperature for 24 h, then transferred to 30% sucrose solution for cryoprotection prior to HE staining.

### 2.6 ELISA for neurotransmitters and inflammatory cytokines

Levels of 5-HT, DA, HVA, NE, IL-1*β*, and IL-6 in serum and striatum were measured using commercial ELISA kits, following the manufacturers’ protocols:

#### 2.6.1 Serum samples

Thawed serum was used directly.

#### 2.6.2 Striatal samples

100 mg of frozen striatal tissue was homogenized in 1 mL of ice-cold physiological saline using a tissue homogenizer (3 cycles of 30 s at 1000 rpm). The homogenate was centrifuged at 5000×g for 15 min at 4 °C, and the supernatant was collected for analysis.

Absorbance was measured at 450 nm using a microplate reader (Bio-Rad, Hercules, CA, USA). Concentrations of target molecules were calculated using standard curves generated for each kit, and results were expressed as ng/mL (serum) or ng/g tissue (striatum).

### 2.7 Striatal hematoxylin-eosin staining

Fixed striatal tissues were processed for HE staining to evaluate pathological changes (1): Tissues were dehydrated through a graded ethanol series (70%, 80%, 90%, 95%, 100%), cleared in xylene, and embedded in paraffin (2); 4-5 μm-thick coronal sections were cut using a microtome (Leica, Wetzlar, Germany) and mounted on polylysine-coated slides (3); Sections were deparaffinized in xylene, rehydrated through a reversed ethanol series, stained with hematoxylin and eosin, dehydrated again, cleared in xylene, and mounted with neutral balsam (4); Stained sections were observed under a light microscope (Olympus, Tokyo, Japan) at 200× and 400× magnification: (i) number of viable neurons; (ii) morphology of dendrites and axons; (iii) integrity of striatal tissue structure; (iv) clarity of nuclear membranes and fullness of cytoplasm; (v) presence of inflammatory cell infiltration.

### 2.8 Serum metabolomics analysis

Serum samples from CON, MOD, and CMXF-H groups (n=10 per group) were analyzed using ultra-high performance liquid chromatography-quadrupole time-of-flight mass spectrometry (UHPLC-Q-Exactive LC-MS; Thermo Fisher Scientific, Waltham, MA, USA) to identify differential metabolites.

#### 2.8.1 Sample pre-treatment

100 μL of serum was mixed with 400 μL of ice-cold methanol (containing 0.1% formic acid) to precipitate proteins. The mixture was vortexed for 30 s, incubated on ice for 10 min, and centrifuged at 12000×g for 15 min at 4 °C. The supernatant was filtered through a 0.22 μm organic phase filter and stored at -80 °C until analysis.

#### 2.8.2 UHPLC conditions

Separation was performed on an Acquity UPLC HSS T3 column (2.1×100 mm, 1.8 μm; Waters, Milford, MA, USA). Mobile phase A: 0.1% formic acid in water; mobile phase B: 0.1% formic acid in acetonitrile. Gradient elution program: 0–2 min, 5% B; 2–10 min, 5%–95% B; 10–12 min, 95% B; 12–12.1 min, 95%–5% B; 12.1–15 min, 5% B. Flow rate: 0.3 mL/min; column temperature: 40 °C; injection volume: 5 μL.

#### 2.8.3 MS conditions

Electrospray ionization (ESI) source, positive/negative ion modes. Full scan range: m/z 50–1000; resolution: 70,000 (full scan) and 17,500 (MS/MS); collision energy: 20, 40, 60 eV. Data were acquired using Xcalibur software (Thermo Fisher Scientific);

#### 2.8.4 Data processing

Raw data were preprocessed (peak alignment, filtering, normalization) using Compound Discoverer 3.1 (Thermo Fisher Scientific). Principal component analysis (PCA) and orthogonal partial least squares-discriminant analysis (OPLS-DA) were performed to identify differential metabolites (VIP >1 and *P* < 0.05).

### 2.9 Quantitative real-time PCR for mRNA expression

Total RNA was extracted from frozen striatal tissues (CON, MOD, CMXF-H groups; n=3 per group) using TRIzol reagent (Invitrogen, Carlsbad, CA, USA), following the manufacturer’s protocol (1): RNA purity and concentration were determined using a NanoDrop 2000 ultra-micro spectrophotometer (Thermo Fisher Scientific) (A260/A280 ratio: 1.8–2.0 indicates high purity) (2); First-strand cDNA was synthesized from 1 μg of total RNA using a PrimeScript RT Reagent Kit with gDNA Eraser (TaKaRa, Dalian, China) to eliminate genomic DNA contamination (3); RT-qPCR was performed on a StepOnePlus Real-Time PCR System (Applied Biosystems, Foster City, CA, USA) using SYBR Premix Ex Taq II (TaKaRa). The reaction system (20 μL) included: 10 μL of SYBR Premix Ex Taq II, 0.4 μL of forward primer (10 μM), 0.4 μL of reverse primer (10 μM), 2 μL of cDNA template, and 7.2 μL of RNase-free water. Cycling conditions: 95 °C for 30 s; 40 cycles of 95 °C for 5 s and 60 °C for 30 s; followed by a melting curve analysis (60 °C to 95 °C) to confirm primer specificity (4); Primer sequences for target genes (DRD1, DRD2, COMT, MAO-B) and the reference gene (GAPDH, used for normalization) are listed in **Table 1**. Relative mRNA expression levels were calculated using the 2^-ΔΔCt^ method.

**Table 1:** Primer information table for DRD1, DRD2, COMT, MAO-B, and GAPDH.

Primer name	Primer sequence
DRD1-Forward primer	5’-TCCAGGGGTTTTGGGAGAAGT-3’
DRD1-Reverse primer	5’-CATCCGCTGGTCCCTAGATTC-3’
GAPDH-Forward primer	5’-TCAAGGCTGAGAACGGGAAG-3’
GAPDH-Reverse primer	5’-TCGCCCCACTTGATTTTGGA-3’
DRD2-Forward primer	5’-ATCGTCTCGTTCTACGTGCC-3’
DRD2-Reverse primer	5’-CAGCATCCTTGAGTGGTGTCT-3’
COMT-Forward primer	5’-ATGCAGAGTGACCACATGAGC-3’
COMT-Reverse primer	5’-TCTTTAGAGGAGGCAGGTCCA-3’
MAO-B-Forward primer	5’-CTGCAGCCCGTCCATTATGA-3’
MAO-B-Reverse primer	5’-GTGTGAGGCTGTTTCAGTGC-3’

### 2.10 Western blotting analysis for protein expression

Protein expression of DRD1, DRD2, COMT, and MAO-B in striatal tissues (CON, MOD, CMXF-H groups; n=3 per group) was detected by Western blotting analysis(WB).

#### 2.10.1 Protein extraction

Frozen striatal tissue was homogenized in ice-cold RIPA lysis buffer (Beyotime, Shanghai, China) containing 1% phenylmethylsulfonyl fluoride (PMSF; 100:1 v/v) to inhibit protein degradation. The homogenate was incubated on ice for 30 min and centrifuged at 12000×g for 15 min at 4 °C. The supernatant (total protein) was collected.

#### 2.10.2 Protein quantification

Protein concentration was determined using a BCA Protein Assay Kit (Beyotime) with bovine serum albumin (BSA) as the standard.

#### 2.10.3 SDS-page and transfer

30 μg of total protein per sample was mixed with 5×SDS loading buffer, boiled for 5 min, separated by 10% SDS-PAGE gel (80 V for stacking gel, 120 V for resolving gel), and transferred to polyvinylidene fluoride (PVDF) membranes (Millipore, Billerica, MA, USA) at 200 mA for 60 min.

#### 2.10.4 Immunoblotting

Membranes were blocked with 5% non-fat milk in Tris-buffered saline with Tween 20 (TBST) for 1 h at room temperature, then incubated overnight at 4 °C with primary antibodies: anti-DRD1 (1:1000), anti-DRD2 (1:1000), anti-COMT (1:1000), anti-MAO-B (1:1000), and anti-β-actin (1:5000, internal reference; all antibodies from Abcam, Cambridge, UK). After washing 3 times with TBST (10 min each), membranes were incubated with horseradish peroxidase (HRP)-conjugated goat anti-rabbit secondary antibody (1:5000; Beyotime) for 1 h at room temperature. Membranes were washed again 3 times with TBST.

#### 2.10.5 Signal detection and quantification

Protein bands were visualized using an enhanced chemiluminescence (ECL) detection kit (Millipore) and imaged with a ChemiDoc XRS+ imaging system (Bio-Rad). Band intensity was quantified using ImageJ software (NIH, Bethesda, MD, USA). Relative protein expression levels were normalized to β-actin.

#### 2.11 Statistical analysis

All experimental data were processed and visualized using GraphPad Prism 8.0 software (GraphPad Software, LLC, USA). Measurement data are expressed as mean ± standard deviation (SD). Repeated measurements were analyzed by repeated measures ANOVA. The remaining data were analyzed by one-way ANOVA. The least significant difference method was used for the *post-hoc* analysis. When the data were not normally distributed, nonparametric tests were used for the item-by-item statistical analysis. F>1 indicates that the variation between groups is larger than the variation within groups, suggesting that the treatment may have an effect on the measured index. *P* < 0.05 was considered statistically significant.

## 3 Results

### 3.1 Chemical constituents of CMXF

**Figure 1:**
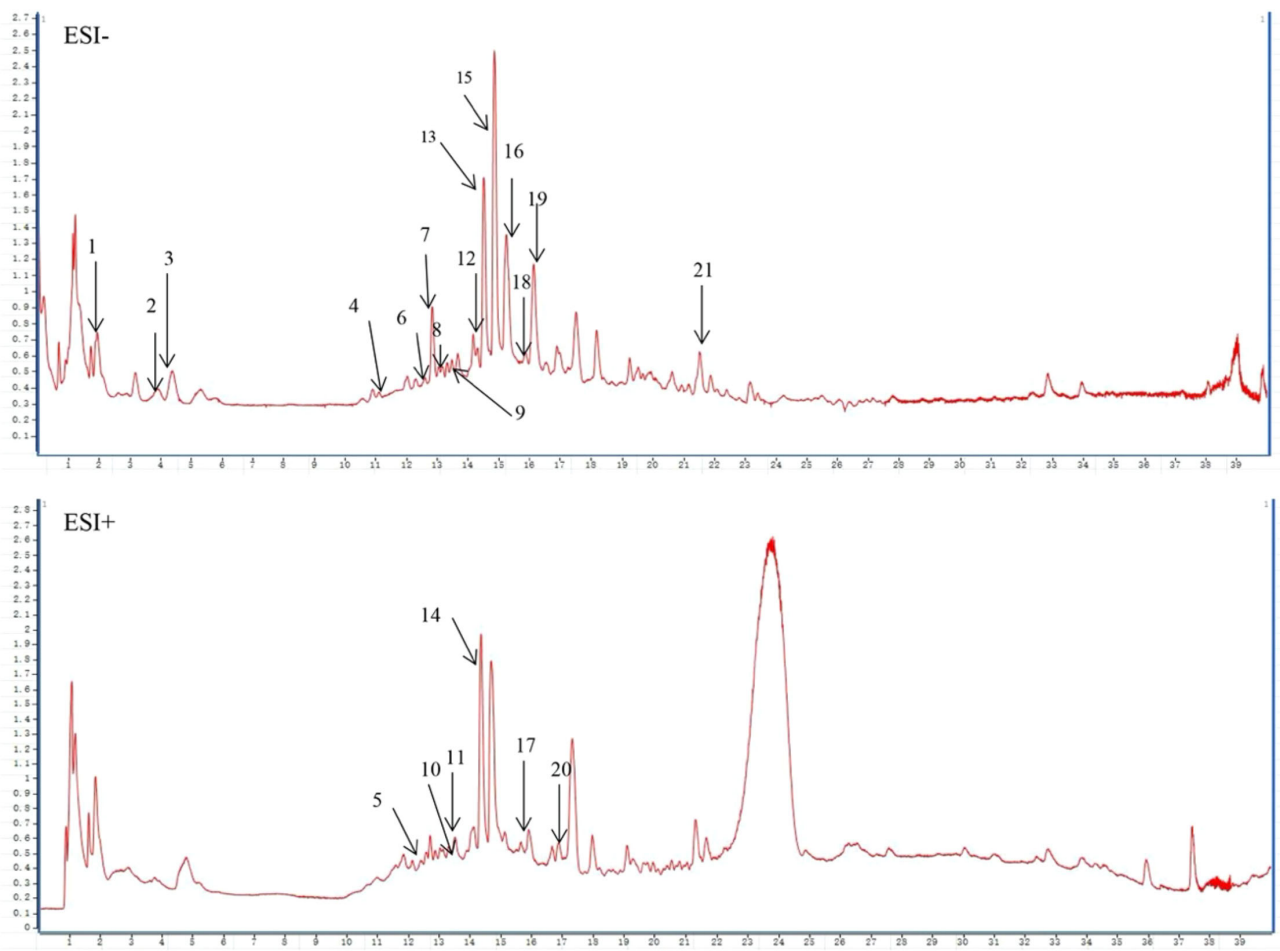
Mass spectrum of CMXF.

The chemical constituents of CMXF were analyzed and identified by UPLC-Q-TOF-MS/MS. Ultimately, a total of twenty-one components, including gallic acid, gastrodin, catechin, sibiricose A3, paeoniflorin, parishin B, and tenuifoliside B, were identified in CMXF. The mass spectrum of CMXF and detailed information for each peak were presented in **Figure 1** and **Table 2**, respectively.

**Table 2:** The chemical components identification of CMXF solution sample based on UPLC/Q-TOF-MS/MS.

No.	ESI	Identifified compounds	Rt (min)	Molecular formula	Observed	MS/MS
1	-	8-debenzoylpaeoniflorin	1.896	C_16_H_24_O_10_	399.1262	341.1830,282.1343,237.0738,185.0423
2	-	Gallic acid	3.804	C_7_H_6_O_5_	169.0142	125.0245,97.0295,79.0190,69.0345
3	-	Gastrodin	4.290	C_13_H_18_O_7_	331.1035	285.0980,161.2459,129.9759,123.0453
4	-	Catechin	11.279	C_15_H_14_O_6_	289.0718	245.0811,205.0507,187.0752,125.0239
5	+	Sibiricose A3	12.391	C_19_H_26_O_13_	485.1266	323.0737,203.0527,185.0417
6	-	Parishin E	12.726	C_19_H_24_O_13_	459.1144	173.0094,129.0194,111.0090
7	-	Oxypaeoniflora	12.893	C_23_H_28_O_12_	495.1508	465.1397,333.0968,281.0660,137.0245
8	-	Methyl gallate	12.995	C_8_H_8_O_5_	183.0299	168.2259,124.0166,78.0108
9	-	Albiflorin	13.265	C_23_H_28_O_11_	525.1615	479.1559,479.1560,121.0295
10	+	Sibiricose A5	13.322	C_22_H_30_O_14_	541.1528	379.1002,361.0898, 203.0526,167.0318
11	+	Sibiricose A6	13.455	C_23_H_32_O_15_	571.1632,549.1581	409.1106,391.1003,233.1650,162.0909
12	-	Paeoniflorin Paeoniflorin	14.179	C_23_H_28_O_11_	525.1614	449.1458,327.1085,165.0556,121.0295
13	-	Parishin B	14.424	C_32_H_40_O_19_	727.2091	483.1103,377.0683,215.0161,107.0492
14	+	Sibiricose A1	14.224	C_23_H_32_O_15_	571.1632,549.1581	409.1110,391.0984,203.0534
15	-	Parishin C	14.892	C_32_H_40_O_19_	727.2091	483.1109,377.0684,215.0166,107.0490
16	-	Parishin A	15.188	C_45_H_56_O_25_	995.3038	751.2033,483.0172,215.0168
17	+	Tenuifoliside B	15.589	C_30_H_36_O_17_	691.1845	409.1096,391.0996,323.0747,167.0309
18	-	1,2,3,4,6-O-Pentagalloylglucose	15.883	C_41_H_32_O_26_	939.1109	769.2758,617.0646,447.0509,169.0126
19	-	galloylpaeoniflorin	15.890	C_30_H_32_O_15_	631.1668	465.1373,313.0535,271.0430,169.0113
20	+	3, 6′-Disinapoyl Sucrose	16.887	C_34_H_42_O_19_	777.2213	409.1109,391.1005,167.0310
21	-	benzoylpaeoniflorin	21.317	C_30_H_32_O_12_	629.1878	583.1811,553.1713,121.0294

### 3.2 Effects of CMXF on behavioral performance in TS mice

**Figure 2:**
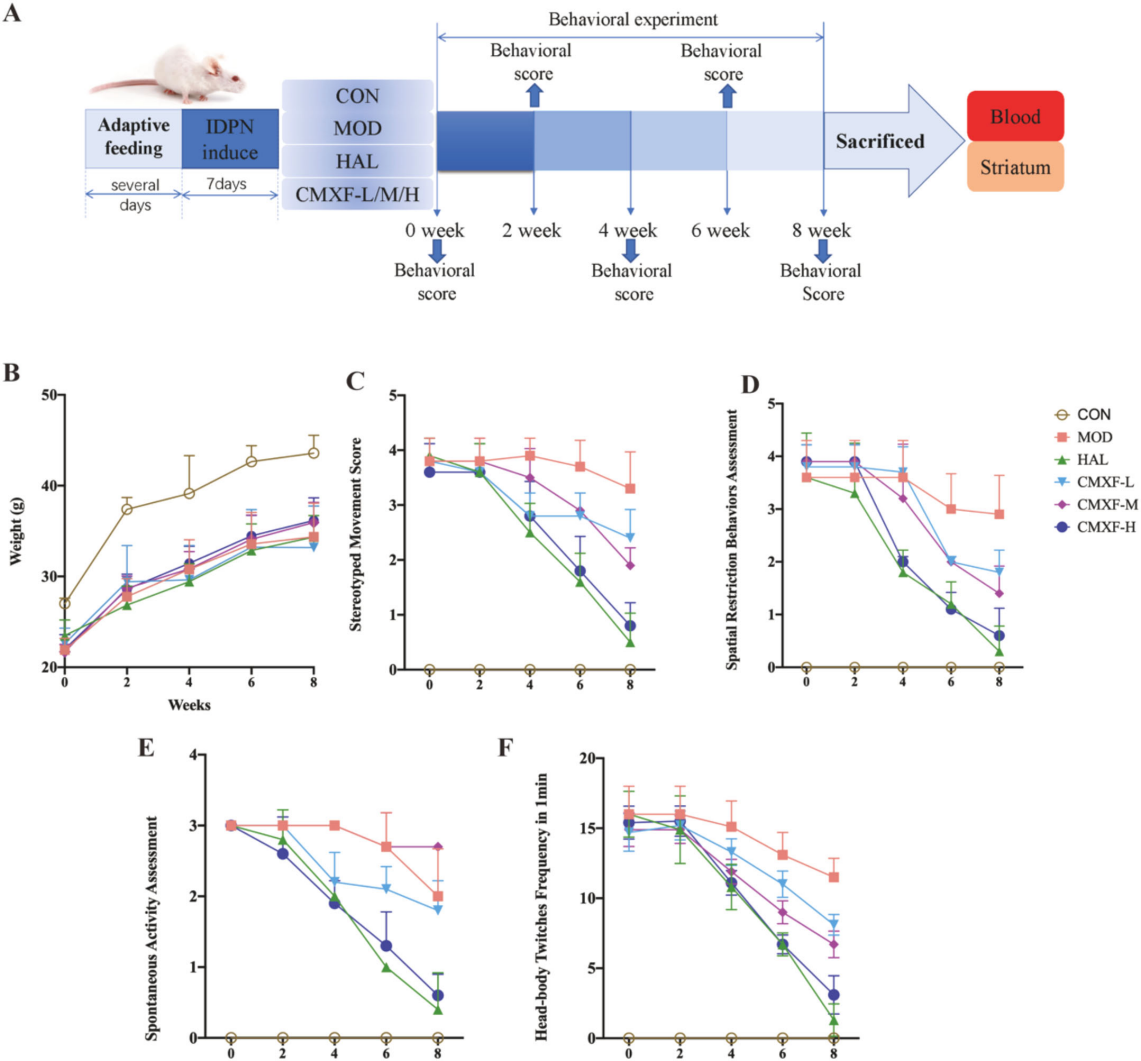
Experimental timeline and behavioral assessments. **(A)** Animal administration process; **(B)** Animal weight; **(C)**Behavioral experiment result of stereotyped movement scoring; **(D)** Behavioral experiment result of spontaneous activity assessment; **(E)** Behavioral experiment result of spatial restriction behaviors assessment; **(F)** Behavioral experiment result of head-body twitches frequency in 1min. The data were expressed as the means ± SD (n = 10).

Behavioral assessments (including scoring of stereotyped movements, spontaneous activity, and spatial restriction behaviors, as well as recording of head-body twitch frequency within 1 minute) were performed at weeks 0, 2, 4, 6, and 8 post-administration to evaluate the therapeutic effects of CMXF on TS mice (**Figure 2B**). During the first 2 weeks of treatment, no significant differences in all behavioral indicators were detected among the HAL, CMXF-L, CMXF-M, CMXF-H, and MOD group. However, from week 4 to week 8 of administration, the HAL group and CMXF-H group exhibited significantly improved behavioral performance compared with the MOD group (*F* > 1, *P* < 0.001). Notably, the improvement of behavioral indices in CMXF-treated groups exhibited a dose-dependent trend, suggesting that CMXF may exert a therapeutic effect on TS in a dose-related manner.

### 3.3 Effects of CMXF on neurotransmitter and inflammatory cytokine levels in serum and striatum of TS mice

ELISA was used to detect the levels of key neurotransmitters 5-HT, DA, HVA, NE and inflammatory cytokines (IL-1*β*, IL-6) in serum and striatum of mice after 8 weeks of treatment.

**Figure 3:**
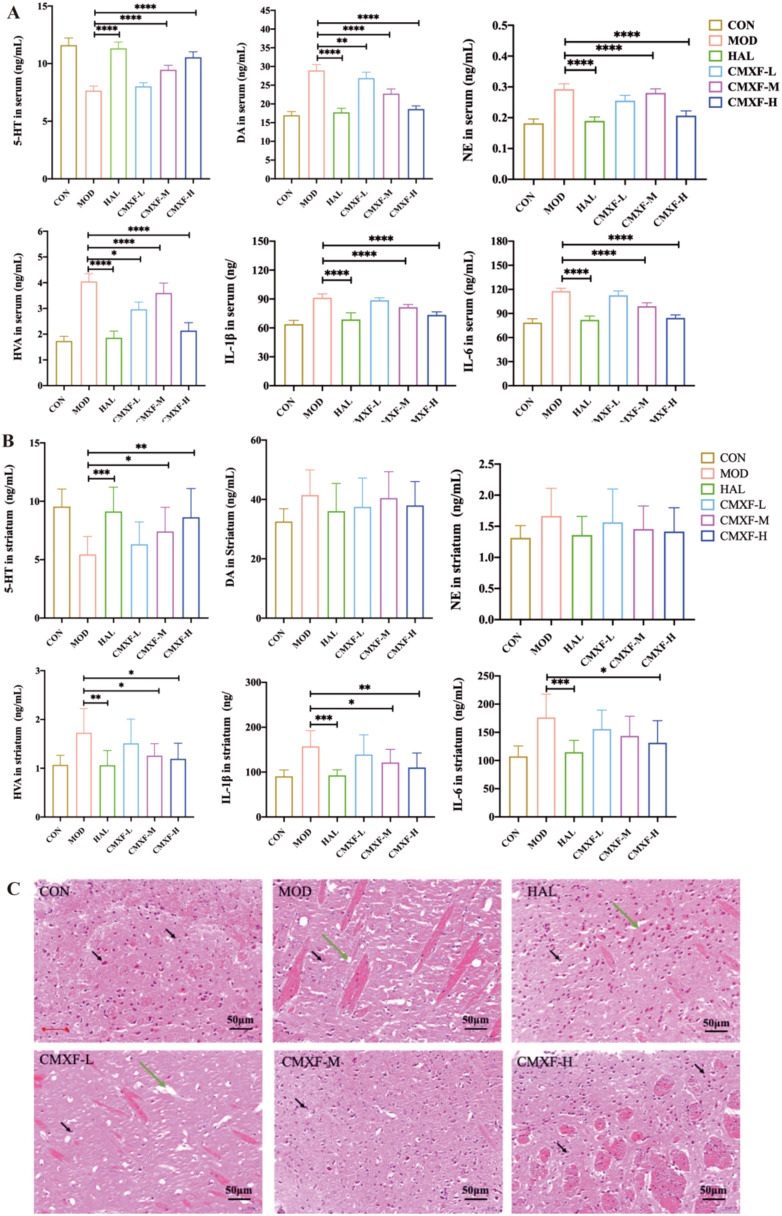
Effects of CMXF on neurotransmitter/cytokine levels and striatal pathology. **(A)** Serum and **(B)** striatal levels of 5-HT, DA, NE, HVA, IL-1*β*, and IL-6 were determined by ELISA. **(C)** Representative HE-stained images of striatal tissues from different groups. Data are expressed as the mean ± SD (n = 10). (Comparing with the model group, *P < 0.05, **P < 0.01, ***P < 0.001, and **** *P* < 0.0001.).

In serum (**Figure 3A**), compared with the MOD group, the HAL group, CMXF-M group, and CMXF-H group showed significant differences in the levels of all detected indicators (5-HT, DA, HVA, NE, IL-1β, IL-6) (*F* > 1, *P* < 0.0001). The CMXF-L group only exhibited significant differences in DA and HVA levels (*F* > 1, *P* < 0.05).

In the striatum (**Figure 3B**), compared with the MOD group, the HAL group showed significant differences in the levels of 5-HT, HVA, IL-1*β*, and IL-6 (*F* > 1, *P* < 0.05); Among the CMXF dose groups, only the CMXF-H group presented significant differences in 5-HT, HVA, IL-1*β*, and IL-6 levels (*F* > 1, *P* < 0.05); No significant differences in all detected indicators were observed between the CMXF-M group and CMXF-H group.

Collectively, these results indicated that CMXF-H could significantly upregulate 5-HT levels in serum and striatum (*F* > 1, *P* < 0.001) while downregulating serum levels of DA, HVA, NE, IL-1*β*, and IL-6 (*F* > 1, *P* < 0.001), implying that CMXF may modulate neurotransmitter balance and inhibit inflammatory responses in TS mice.

### 3.4 Effects of CMXF on the striatal HE staining in TS mice

The HE staining results were shown in the **Figure 3C**. In the CON group, the striatal tissue exhibited intact structure with no neuronal necrosis; neurons had clear nuclear membranes and abundant cytoplasm. In the MOD group, the striatum showed extensive neuronal necrosis (black arrows), accompanied by karyopyknosis, hyperchromasia, and vacuolar structures (green arrows). The CMXF-H group showed restored striatal structure with minimal neuronal damage and no vacuolar structures. It indicated that the high dose of CMXF was effective in alleviating striatal pathological damage in TS mice.

### 3.5 Effects of CMXF on mRNA and protein expressions of DRD1, DRD2, COMT, and MAO-B in striatum of TS mice

**Figure 4:**
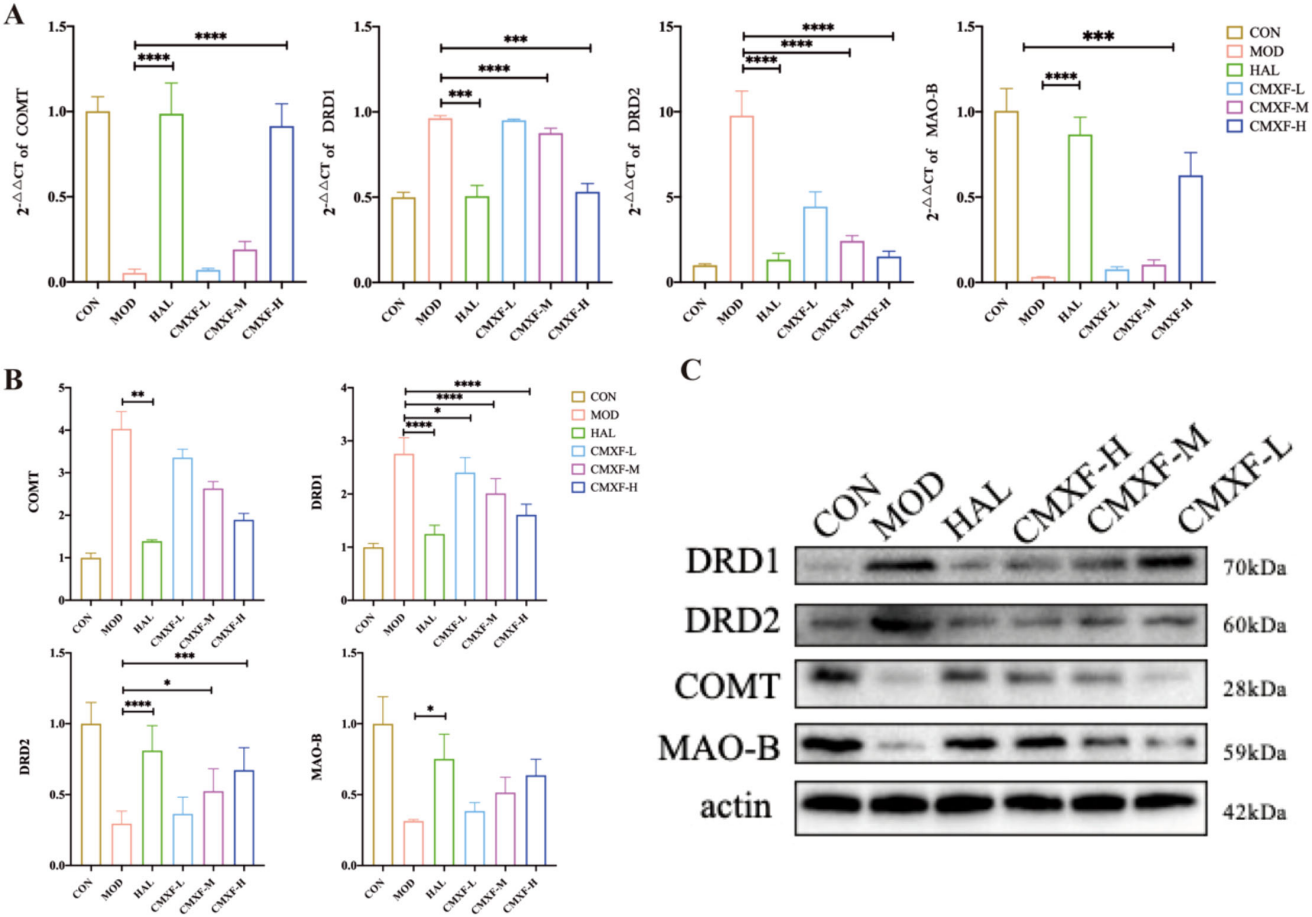
**(A)** CMXF regulates the mRNA and protein expression of dopamine signaling molecules in the striatum. **(A)** Relative mRNA levels of DRD1, DRD2, COMT, and MAO-B determined by RT-qPCR. **(B)** Quantitative analysis and **(C)** representative images of protein levels detected by Western blot. Data are expressed as the mean ± SD (n = 3). (Comparing with the model group, *P < 0.05, **P < 0.01, ***P < 0.001, and **** *P* < 0.0001.).

Statistical analysis of RT-qPCR revealed significant differences in the mRNA expressions of DRD1, DRD2, COMT, and MAO-B between the MOD group, HAL group, and CMXF-M group (*F* > 1, *P* < 0.0001). After pharmacological intervention, the mRNA expressions of these four molecules showed a consistent upward trend, indicating that CMXF may regulate the transcription of genes related to dopamine metabolism (**Figure 4A**).

After 8 weeks of treatment, significant differences in the protein levels of DRD1, DRD2, COMT, and MAO-B were observed between the MOD group and HAL group (*P* < 0.05); In the CMXF-treated groups, only the protein expressions of DRD2 and MAO-B were significantly altered compared with the MOD group (*F* > 1, *P* < 0.05), while the protein levels of COMT and DRD1 in the CMXF-H group remained unchanged; Compared with the CON group, the protein expressions of all detected molecules in the MOD group showed significant differences (*F* > 1, *P* < 0.0001), and the expression patterns in the HAL group and CMXF-H group were consistent with those of the CON group (**Figure 4B, C**).

These results implied that CMXF may modulate dopamine signaling by inhibiting the protein expressions of DRD1/DRD2 and inducing the protein expression of MAO-B, while its regulation of COMT may mainly occur at the transcriptional level.

### 3.6 Effects of CMXF on serum metabolomics profiles in TS mice

To explore the impact of CMXF on serum metabolism in TS mice, serum metabolic profiles of TS model mice pre- and post-administration were analyzed using multivariate statistical methods and metabolite functional annotation, with a focus on the CON, MOD, and CMXF-H group.

**Figure 5:**
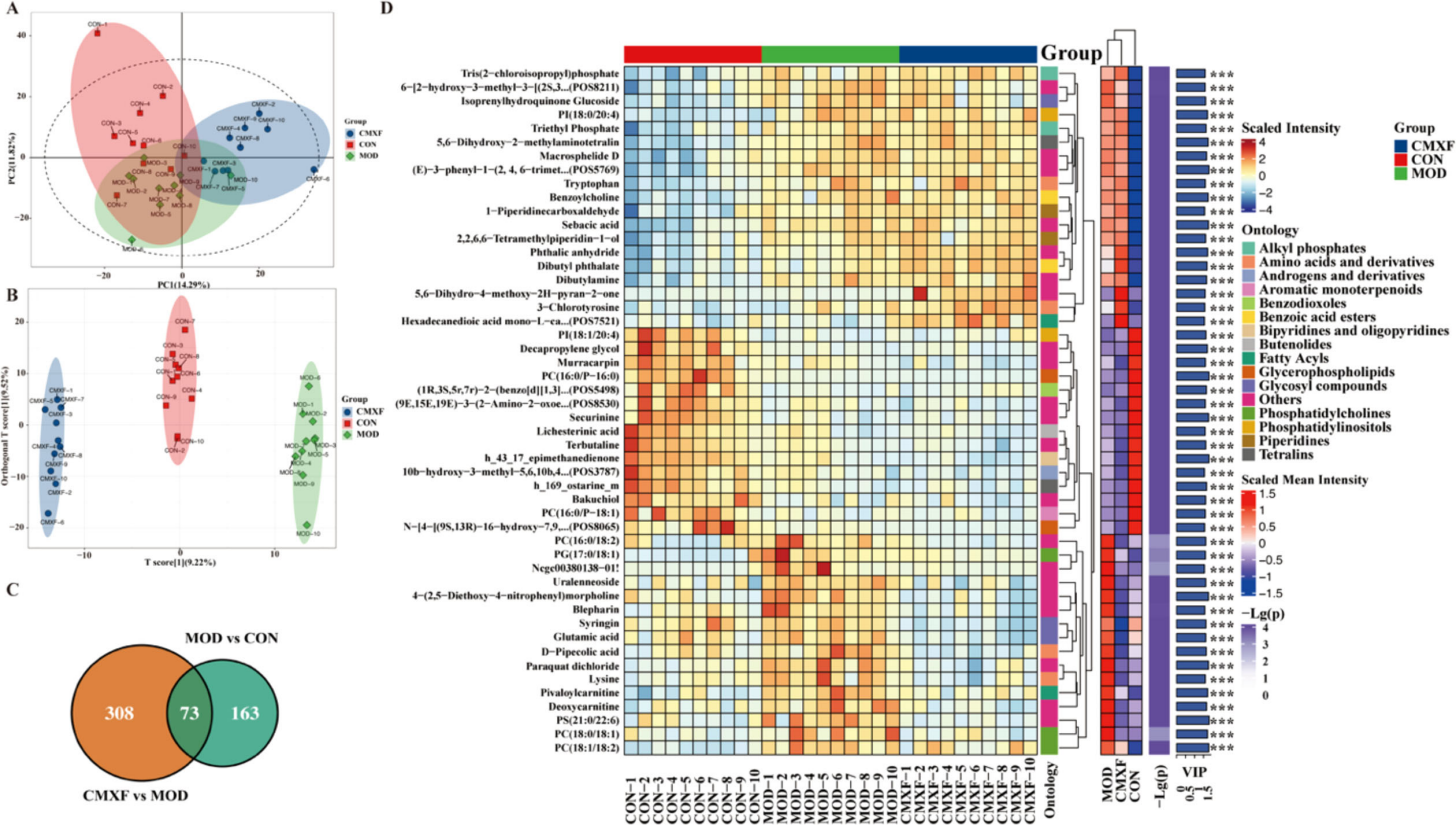
Serum metabolomics profiling and differential metabolite analysis. **(A)** Principal component analysis (PCA) and **(B)** orthogonal partial least squares-discriminant analysis (OPLS-DA) of serum metabolites from CON, MOD, and CMXF groups. **(C)** Venn diagram illustrating common and unique differential metabolites. **(D)** Hierarchical clustering heatmap of the top 50 differential metabolites ranked by VIP scores.

Serum metabolic profiles were first evaluated via PCA and OPLS-DA to identify differential metabolites. Unsupervised PCA revealed partial separation of serum metabolic profiles among the CON, MOD, and CMXF-H groups, though the distinctions between the groups were not definitive (**Figure 5A**). In contrast, supervised OPLS-DA exhibited clear demarcations in serum metabolic profiles across the three groups, indicating statistically significant differences in metabolite composition. A permutation test was further conducted to validate the reliability of the OPLS-DA model; the Q2 regression line intersected the vertical axis below zero, confirming the absence of overfitting and supporting the validity of subsequent differential metabolite analysis (**Figure 5B**). Venn analysis was employed to characterize common and unique differential metabolites across comparison pairs (MOD vs CON, CMXF-H vs MOD), which identified 73 shared differential metabolites (**Figure 5C**). Additionally, the top 50 differential metabolites, ranked by variable importance in projection (VIP) values, were selected to construct a hierarchical clustering heatmap, facilitating the visualization of distinct metabolite expression patterns among the three groups (**Figure 5D**).

**Figure 6:**
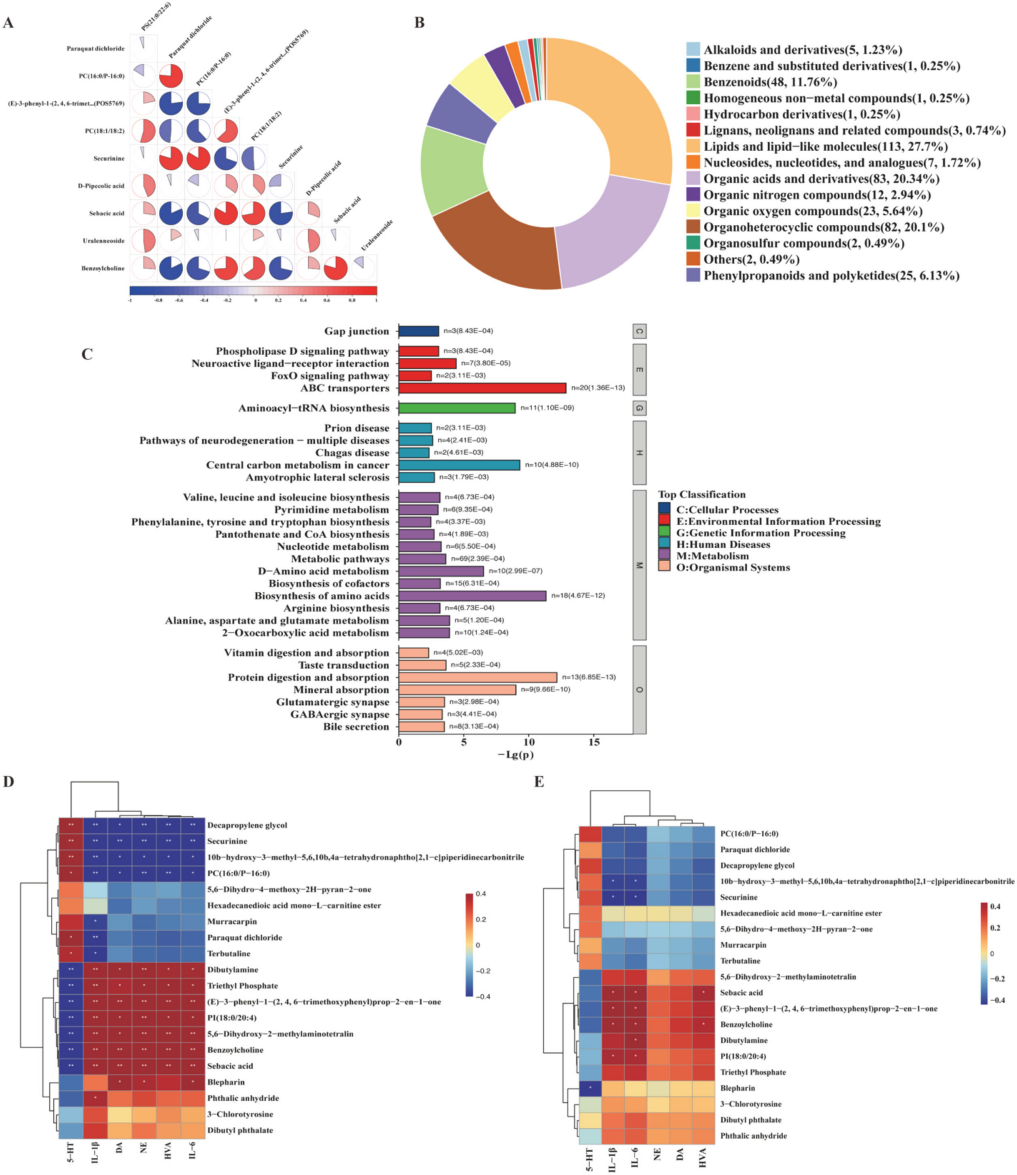
Functional annotation of differential metabolites and their correlation with key neurotransmitters and cytokines. **(A)** Correlation network among the top 10 VIP metabolites. **(B)** HMDB super class classification of the identified metabolites. **(C)** KEGG pathway enrichment analysis (top 30 pathways). **(D, E)** Correlation heatmaps between the top 20 VIP metabolites and the levels of 5-HT, IL-1*β*, NE, DA, and HVA in serum **(D)** and striatum **(E)**.

To further elucidate the properties of these differential metabolites, Pearson correlation analysis was performed to calculate correlation coefficients between significantly different metabolites; results were presented as a correlation coefficient matrix heatmap (**Figure 6A**). The proportion of the colored interval in the heatmap reflected the strength of the correlation, with larger proportions indicating stronger positive or negative correlations. For functional classification, differential metabolites were analyzed using the Human Metabolome Database (HMDB) super class method based on their structural and functional characteristics, and results were visualized as a donut chart (**Figure 6B**). This classification revealed 15 distinct metabolite categories, among which lipids and lipid-like molecules (n=113), organic acids (n=83), organoheterocyclic compounds (n=82), aromatic compounds (n=48), and organic oxygen compounds (n=23) were the most prominent. Furthermore, Kyoto Encyclopedia of Genes and Genomes (KEGG) pathway analysis was conducted to annotate the biological functions of the differential metabolites, with the top 30 enriched pathways visualized (**Figure 6C**). The analysis identified seven major KEGG pathway categories: Metabolism, Genetic Information Processing, Environmental Information Processing, Cellular Processes, Organismal Systems, and Human Diseases (enriched in neurodegenerative diseases)”, providing insights into the potential metabolic pathways modulated by CMXF.

### 3.7 Correlation analysis between key biological indicators and differential metabolites in TS mice

To explore the potential associations between key neurotransmitters, inflammatory cytokines, and differential metabolites in TS mice, Pearson correlation analysis was conducted to assess the relationships between the levels of 5-HT, IL-1*β*, NE, DA, and HVA in serum and striatum, and the top 20 differential metabolites ranked by variable VIP values. The correlation results were visualized as heatmaps (**Figure 6D** for serum indicators; **Figure 6E** for striatal indicators).

In serum, specific differential metabolites exhibited distinct correlation patterns with the aforementioned biological indicators: Dibutylamine, triethyl phosphate, (E)-3-phenyl-1-(2, 4, 6-trimethoxyphenyl)prop-2-en-1-one, phosphatidylinositol (18:0/20:4) [PI(18:0/20:4)], 5,6-dihydroxy-2-methylaminotetralin, benzoylcholine, and sebacic acid showed a positive correlation with serum IL-1*β*, NE, DA, HVA, and IL-6. In contrast, decapropylene glycol, securinine, and phosphatidylcholine (16:0/P-16:0) [PC (16:0/P-16:0)] exhibited a negative correlation with the same set of serum indicators (IL-1*β*, NE, DA, HVA, IL-6).

Correlation trends between these differential metabolites and the corresponding indicators in the striatum were generally consistent with those observed in serum, though minor variations in correlation strength were noted (**Figure 6E**). These findings suggest potential links between the metabolic alterations induced by CMXF and the regulation of neurotransmitter balance and inflammatory responses in TS mice.

## 4 Discussion

### 4.1 Potential roles of CMXF’s herbal components in alleviating TS-associated impairments

CMXF, a TCM formulation for TS, comprises multiple herbal components with documented neuroprotective and neuroregulatory properties. For instance, *Gastrodia elata* (19), *Acorus tatarinowii* (20), and *Uncaria* (21)—key components of CMXF—are widely recognized to promote cerebral blood circulation and metabolism, which may indirectly support intellectual development in children with TS. A previous study showed that gastrodin could inhibit GSK-3*β* phosphorylation, regulate SERT expression, and influence 5-HT levels, thereby suppressing DA release in the striatum. It also mitigates striatal inflammation and oxidative stress via the Nrf-2/HO-1/HMGB1/NF-*κ*B pathway (22). The chemical components identification of CMXF solution sample based on UPLC/Q-TOF-MS/MS also showed that there were some components such as gastrodin, parishin A/B/C/E, and paeoniflorin, which was the same as the previous study. Optimal cerebral blood flow and metabolic activity are essential for maintaining normal brain function, and this improvement could potentially enhance attention and cognitive abilities in affected individuals, as cognitive deficits are often comorbid with TS.

Additionally, other herbs in CMXF, such as *Poria* and *Polygala* (23), have been reported to regulate the functional state of the cerebral cortex and improve sleep quality. Insomnia and sleep disturbances are common in children with TS, and adequate sleep is critical for brain development and functional recovery in pediatric populations. By mitigating sleep-related issues, these components may indirectly contribute to the alleviation of TS symptoms, highlighting the holistic approach of CMXF in addressing both core tics and associated comorbidities.

### 4.2 CMXF modulates neurotransmitter homeostasis, inflammatory responses, and striatal pathological damage

A growing body of evidence suggests that TS pathogenesis is closely linked to neurotransmitter imbalances, particularly of DA, NE, and 5-HT (24). Patients with TS often exhibit either hyperactivation or relative insufficiency of 5-HT function: hyperactivated 5-HT may excessively inhibit other neurotransmitter systems, disrupting brain information transmission, while insufficient 5-HT fails to regulate the nervous system effectively—both scenarios can trigger or exacerbate TS symptoms (24). DA, as a key monoamine neurotransmitter, plays a central role in TS: dysfunction in mesocortical, mesolimbic, and striatal DA pathways is associated with cognitive deficits and tic manifestation (25).

Consistent with this, our previous studies (26) demonstrated that CMXF altered the levels of DA, 5-HT, HVA (a DA metabolite), and NE in TS model mice. The present study extends these findings by showing that high-dose CMXF significantly increases 5-HT levels in serum and striatum, while decreasing serum DA, HVA, NE, and proinflammatory cytokines (IL-1β, IL-6). IL-1β and IL-6 (27) are known to participate in neuroinflammatory processes that impair neural development and function, suggesting that CMXF may also exert anti-TS effects by suppressing neuroinflammation.

Furthermore, HE staining confirmed that CMXF-H alleviates striatal pathological damage in TS mice—including reducing neuronal necrosis and vacuolar structures—compared to the model group. The striatum (28) is a critical brain region involved in motor control and tic regulation; thus, the restoration of striatal structure by CMXF provides histological evidence for its therapeutic effect, while also highlighting the striatum as a potential target for TS intervention.

### 4.3 CMXF regulates TS-associated metabolic pathways: lipid and organic acid metabolism

Serum metabolomic analysis revealed that CMXF modulates multiple differential metabolites, with prominent enrichment in lipid metabolism and organic acid metabolism pathways. Lipids are essential components of cell membranes and play key roles in signal transduction and energy metabolism—processes critical for normal neuronal function. For example, metabolites such as tris(2-chloroisopropyl) phosphate, triethyl phosphate, and phosphatidylinositol (PI (18:1/20:4))—identified in our study—are closely linked to lipid metabolism. Abnormalities in lipids and lipid-like molecules may disrupt neurotransmitter synthesis, release, or metabolism (29), which could contribute to TS pathogenesis; thus, CMXF-induced normalization of lipid metabolism may indirectly restore neurotransmitter balance.

Organic acids, such as sebacic acid, 3-chlorotyrosine, and 5,6-dihydro-4-methoxy-2H-pyran-2-one, are involved in energy metabolism, substance synthesis, and cellular signaling. Pediatric populations have immature metabolic systems, making them more susceptible to environmental factors (e.g., diet, drugs, infections) that disrupt organic acid metabolism. Such disruptions may reduce cerebral energy supply, impairing brain function and leading to TS-related symptoms (e.g., inattention, hyperactivity) (29). Additionally, organic acids participate in neurotransmitter metabolism and regulation; abnormal organic acid metabolism could therefore interfere with neurotransmitter homeostasis, further exacerbating TS. By regulating these organic acid metabolites, CMXF may help restore both energy metabolism and neurotransmitter balance in TS.

### 4.4 CMXF targets dopamine-related receptors and metabolic enzymes

The present study demonstrated that CMXF modulates the expression of key molecules involved in DA signaling: it inhibits the expression of DA receptors (DRD1, DRD2) and induces the expression of DA metabolic enzymes (COMT, and MAO-B). These findings align with existing evidence linking these molecules to TS (1): DRD1 is highly expressed in the prefrontal cortex, a region critical for executive function. Reduced DRD1 expression impairs DA signal transmission, which is associated with TS and attention deficit hyperactivity disorder (ADHD) comorbidity (30, 31) (2). A single nucleotide polymorphism (SNP) within the 25 kb sequence of the DRD2 gene has been identified as a genetic risk factor for TS, highlighting DRD2’s role in TS susceptibility (32) (3). COMT is involved in the metabolism of DA, 5-HT, and NE—all neurotransmitters implicated in TS—and has also been associated with ADHD-related behaviors (33) (4). MAO-B participates in the metabolism of NE and DA; MAO-B inhibitors are clinically effective for ADHD (34), suggesting that upregulating MAO-B via CMXF may help normalize DA and NE levels in TS.

By targeting these molecules, CMXF directly modulates DA signaling— a core pathway in TS pathophysiology—further supporting its multi-targeted therapeutic action.

### 4.5 Multi-target, multi-pathway action of CMXF: aligning with TCM’s holistic principles

Collectively, the above findings indicate that CMXF exerts anti-TS effects via a multi-target, multi-pathway mechanism: it regulates neurotransmitter balance (5-HT, DA, NE), suppresses neuroinflammation (IL-1β, IL-6), alleviates striatal pathology, modulates lipid and organic acid metabolism, and targets DA-related receptors/enzymes (DRD1, DRD2, COMT, MAO-B). This aligns with the holistic therapeutic principle of TCM, which emphasizes restoring the body’s overall homeostasis rather than targeting a single pathway.

**Figure 7:**
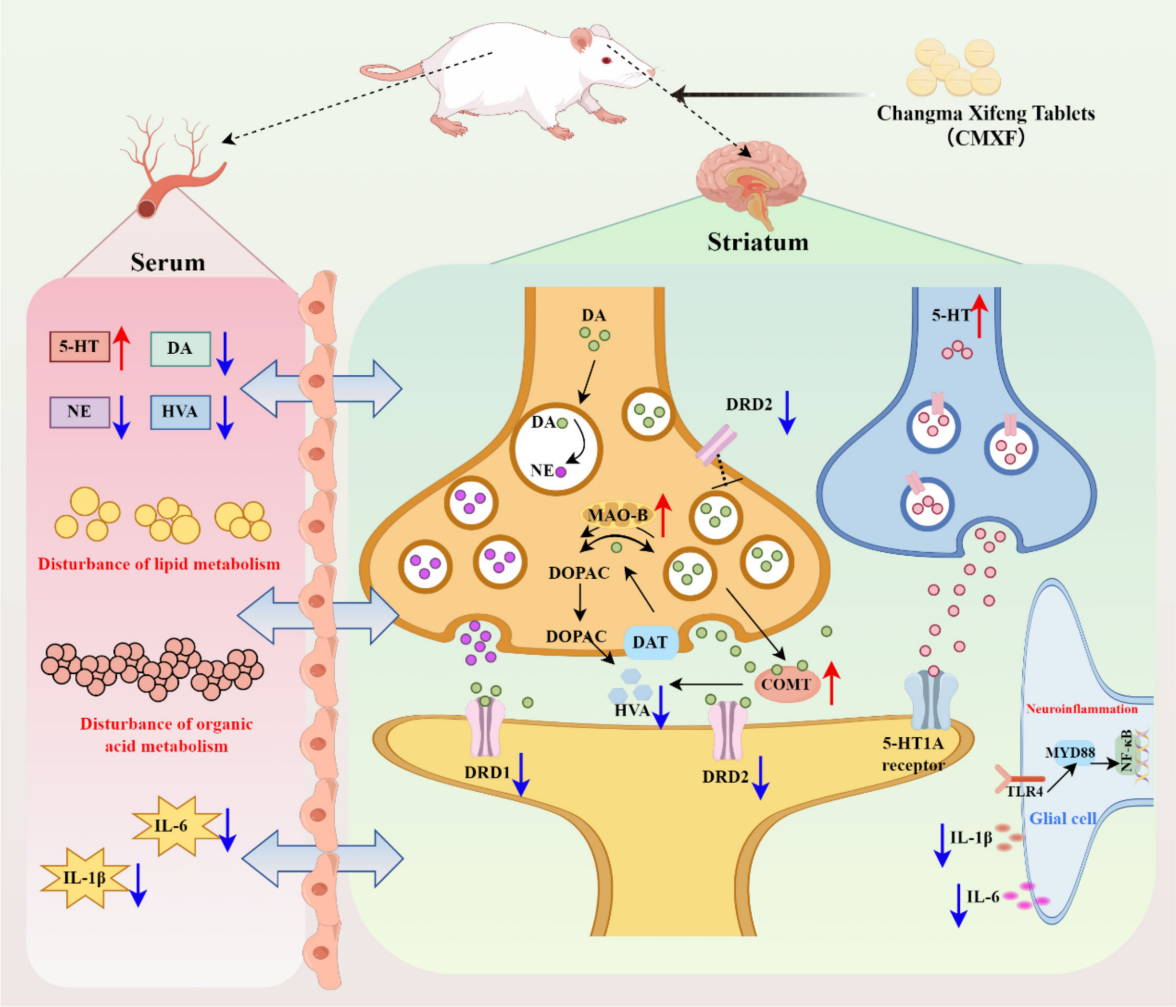
Mechanism of CMXF in alleviating TS. TS model mice were administrated by CMXF. CMXF exerts neurotransmitter balance in serum and striatum(5-HT, DA, NE), suppresses neuroinflammation (IL-1β, IL-6), alleviates striatal pathology, modulates lipid and organic acid metabolism, and targets DA-related receptors/enzymes (DRD1, DRD2, COMT, MAO-B).

Our previous study (26) further supports this: CMXF was shown to regulate the body’s systemic state, improve clinical response rates, and alleviate TS symptoms in patients. Together, these preclinical and preliminary clinical data highlight CMXF’s potential as a safe and effective intervention for TS. The mechanism of CMXF in alleviating TS was shown in **Figure 7**.

### 4.6 Limitations and future directions

Despite these insights, the present study has several limitations. First, the findings are derived from a murine TS model induced by IDPN, which may not fully recapitulate the complexity of human TS, including aspects such as genetic heterogeneity and common comorbidities. Second, although multiple mechanistic pathways were explored, the interactions among these pathways—such as how metabolic alterations influence neurotransmitter dynamics—remain to be fully elucidated. Future studies should address these gaps by employing more clinically relevant TS models, such as genetic models, and by further investigating the cross-talk between metabolic, neurotransmitter, and inflammatory pathways.

## 5 Conclusions

This study demonstrates that CMXF exerts anti-TS effects through multi-target mechanisms involving neurotransmitter regulation, anti-inflammatory activity, striatal tissue protection, and metabolic normalization. Specifically, CMXF treatment increased 5-HT levels in both serum and striatum, while reducing concentrations of DA, HVA, NE, IL-1β, and IL-6 in serum. Pathological damage in the striatum—such as neuronal necrosis and vacuolar structures—was also alleviated. At the molecular level, CMXF downregulated the expression of dopamine receptors DRD1 and DRD2 and upregulated key metabolic enzymes COMT and MAO-B. Serum metabolomics further revealed that CMXF significantly modulated lipid and organic acid metabolism, influencing metabolites such as phosphatidylinositol and sebacic acid. These results highlight the ability of CMXF to restore neurochemical, inflammatory, and metabolic homeostasis in TS, reflecting the holistic interventional strategy characteristic of traditional Chinese medicine. Our findings provide new mechanistic insights into CMXF therapy for TS and suggest promising avenues for developing targeted treatments for neurodevelopmental disorders.

## Data Availability

The original contributions presented in the study are publicly available. This data can be found here: OMIX database, accession OMIX015895 (https://ngdc.cncb.ac.cn/omix/preview/mb2mT482), OMIX015896 (https://ngdc.cncb.ac.cn/omix/preview/J3BPwv8h).
