# Supplementary material for: Mechanisms of Changma Xifeng tablet in alleviating Tourette syndrome via modulation of neurotransmitters, inflammatory responses, and metabolic pathways
Source: Front Psychiatry. 2026 Apr 10;17:1773753. doi: 10.3389/fpsyt.2026.1773753 (PMC13106507; doi:10.3389/fpsyt.2026.1773753)
Supplement: Supplementary file 1 [file Table1.docx]

Mechanisms of Changma Xifeng tablet in alleviating Tourette syndrome via modulation of neurotransmitters, inflammatory responses, and metabolic pathways

Yuanyang Shao^1#^, Chang-e Guo^2#^, Kun Gao^1^, Meng Bian^1^, Lili Li^1^，Lingyan Zhang^1^, Mengjuan Wu^1^, Juan Wang^1*^，Chunsheng Zhu^1*^

1 Department of Chinese Medicine, The First Affiliated Hospital of Zhengzhou University, Zhengzhou, China;

2 Pharmacy Department, Beijing Fengtai Hospital of Chinese Medicine (Beijing Fengtais Hospital of Nanyuan District), Beijing, China;

**Table S1 Gradient elution condition of UPLC-Q-TOF-MS/MS**

| Time（min） | A（%） | B（%） |
| --- | --- | --- |
| 0 | 100 | 0 |
| 2 | 100 | 0 |
| 3 | 70 | 30 |
| 13 | 40 | 60 |
| 23 | 0 | 100 |
| 26 | 0 | 100 |
| 26.1 | 100 | 0 |
| 30 | 100 | 0 |

**Table S2 the source gas parameters**

| Parameter | Set point |
| --- | --- |
| Ionspray Voltage | +5500/-4500V |
| Temperature | 500℃ |
| lon Source Gasl | 60 psi |
| lon Source Gas2 | 60 psi |
| Curtain Gas | 45 psi |
| Declustering Potential | 60V |
| Tof Ms Scan CE | 10V |
| Product lon Scan CE | 40V |
| Collision Energy Spread | 20V |

Table S3 Behavioral assessments scores

| Score | Stereotyped Movement | Spontaneous Activity | Spatial Restriction Behavior |
| --- | --- | --- | --- |
| 0 | None | Quiet or Normal | Novel Object Exploration |
| 1 | Clockwise or counterclockwise discontinuous circling behavior, accompanied by occasional head twitching | Hyperexcitability | Twitch-like response with rapid twitching after stillness |
| 2 | Occasional abnormal head and neck movements, as well as sniffing, licking, and biting behaviors | Increased exploratory behavior | Increased exploratory behavior |
| 3 | Continuous circling, increased body lifting, sniffing, repetitive grooming movements | Running | Upright sniffing and burrowing-like behavior |
| 4 | Increased abnormal horizontal and vertical head and neck movements | Running and jumping | Aggressive behavior with continuous circling |
